# Effect of Addition of Metal Oxide Nanoparticles on the Strength of Heat-Cured Denture Base Resins: Protocol for Systematic Review and Meta-Analysis of In Vitro Studies

**DOI:** 10.2196/59999

**Published:** 2024-09-25

**Authors:** Pragati Kaurani, Amit Hindocha, Amit Porwal, Abhijit Tambe, Carrie Price, Vidhani Goel, Henry Krasner, Jagdish Khubchandani, Kavita Batra

**Affiliations:** 1 Department of Prosthodontics Crown and Bridge Mahatma Gandhi Dental College and Hospital Jaipur India; 2 Department of Prosthodontics and Crown and Bridge Sinhgad Dental College and Hospital Pune India; 3 Department of Prosthetic Dentistry College of Dentistry Jazan University Jazan Saudi Arabia; 4 Department of Prosthodontics Crown and Bridge Sau Mathurabai Bhausaheb Thorat Institute of Dental Sciences and Research Nashik India; 5 Albert S Cook Library Towson University Towson, MD United States; 6 School of Public Health, University of Nevada Las Vegas Las Vegas, NV United States; 7 Kirk Kerkorian School of Medicine at UNLV Las Vegas, NV United States; 8 College of Health, Education, and Social Transformation, New Mexico State University Las Cruces, NM United States; 9 Department of Medical Education Kirk Kerkorian School of Medicine at UNLV Las Vegas, NV United States; 10 Office of Research Kirk Kerkorian School of Medicine at UNLV Las Vegas, NV United States

**Keywords:** nanoparticles, strength, denture base resins, polymethyl methacrylate, denture, dentures, resin, resins, metal oxide

## Abstract

**Background:**

Metal oxide nanoparticle–reinforced polymethyl methacrylate (PMMA) has been shown to improve mechanical properties, such as strength. Different types of metal oxide nanoparticles have been used previously, but the comparative effect on the strength of heat-cured denture base resins remains unclear.

**Objective:**

This is a protocol for a systematic review and meta-analysis that will aim to pool evidence to compare and analyze the effects of the addition of different metal oxide nanoparticles, with varied sizes and concentrations, on the strength (flexural, impact, transverse, compressive tensile strength, and fracture toughness) of heat-cured PMMA. In addition, this review aims to analyze methodological factors, such as adherence to testing and sample-making guidelines, and the effects of surface treatments of the nanoparticles on the strength of heat-cured denture base resins.

**Methods:**

The protocol has been registered in the Open Science Framework. Search strategies to identify studies on the effect of metal oxide nanoparticles on the strength of heat-cured PMMA were developed by the subject matter expert in library science. Following this, a systematic search of 5 electronic databases (PubMed [NCBI], Scopus [Elsevier], Cochrane Library [Wiley], CINAHL Plus with Full Text [EBSCO], and Dimensions Free Web App) was conducted to retrieve in vitro studies published in English from January 2012 to October 2023. Along with this citation chasing, other online sources and gray literature were also searched. Furthermore, papers will be screened, and appropriate data elements will be extracted in a standardized manner. A risk-of-bias assessment will be performed using a modified Cochrane Risk of Bias Tool. A meta-analysis will be performed using a random-effects model.

**Results:**

Search in databases resulted in 1837 papers, of which 1752 were duplicates, leaving 85 records that were screened for titles and abstracts based on the eligibility criteria. A similar search run on other online sources identified 129 papers that will be further analyzed for inclusion. The study was initiated in November 2023 and research questions and search strategies were formulated. The proposed study is expected to be completed by December 2024.

**Conclusions:**

This systematic review will comprehensively analyze the effects of the incorporation of metal oxide nanoparticles in heat-cured denture base resins on the strength of the material. We anticipate gaining a deeper understanding of the effects and method of use of metal oxide nanoparticles to improve the strength of PMMA denture base resins.

**International Registered Report Identifier (IRRID):**

PRR1-10.2196/59999

## Introduction

Acrylic resins used in dentistry are composed of compounds that are natural or artificial in origin and have several repeating structural units or monomers that form the macromolecules or polymers [1]. Although there have been recent technical advances in denture base materials (DBMs), such as computer-aided design and computer-aided manufacturing, heat-cured polymethyl methacrylate (PMMA) continues to remain as one of the most widely used DBMs [2-4]. Even with its prevalent use, it continues to have poor mechanical properties (eg, strength). Heat-cured dentures are known to fracture under repeated occlusal and functional loads [5,6]. For optimum clinical usage and longevity, denture base resins should withstand masticatory forces and, thus, must have good mechanical properties (eg, strength). Overall, dentures are subjected to a combination of tensile, compressive, and shear forces and are susceptible to sudden drop, which may result in denture base fracture [7,8].

Furthermore, DBMs should have high flexural strength to withstand the mastication forces, showing no deformation or fracture [9]. To improve the mechanical properties of PMMA, reinforcement with nanoparticles has been shown to have promising results. Nanoparticles affect the mechanical properties of materials due to their potential to create new and strong bonds and make them more reactive when compared with macro- or microparticles [10]. With the recent advances in nanotechnology, various metal oxide nanoparticles, such as copper oxide (CuO), titanium dioxide (TiO_2_), zirconium dioxide (ZrO_2_), zinc oxide (ZnO), silicon dioxide (SiO_2_), and others, have been developed to enhance the properties and clinical performance of denture base resins [11-18].

Previous research has shown that the properties of nanocomposite can be influenced by factors, such as the type, size, shape, concentration of nanoparticles, and interaction of the nanoparticles with the polymer matrix [17,19-21]. Although few systematic reviews have attempted to analyze the effect of the addition of nanoparticles on the mechanical properties [22-25], they were limited to a single type of metal oxide nanoparticle. Furthermore, while the effect of metal oxide nanoparticles on certain aspects of strength, such as flexural and impact strength, has been reviewed, collective evidence of different types of clinically relevant strengths remains unclear. Therefore, the primary objective of this review is to systematically synthesize the evidence to compare and analyze the effects of the addition of metal oxide nanoparticles on the strength (flexural, impact, transverse, compressive tensile strength, and fracture toughness) of heat-cured PMMA denture resins. The secondary objective is to determine the optimum size and concentration of nanoparticles to provide improved strength of PMMA. In addition, this review also aims to analyze methodological factors, adherence to testing and sample-making guidelines, and surface treatments of nanoparticles on the strength of heat-cured denture base resins. The findings of this review will serve as recommendations for the optimum use of metal oxide nanoparticles to improve the mechanical properties of heat-cured PMMA.

## Methods

### Ethical Considerations

This study is a review of existing in vitro studies, not involving human subjects, and thus an institutional ethical clearance was not necessary.

### Protocol Registration

To have robust methodology and transparency in reporting, the protocol of the review has been registered in the Open Science Framework (OSF) [26]. Open Science Framework is an open and free platform that supports research by helping in protocol registration and collaborations. This study will be conducted per the PRISMA (Preferred Reporting Items for Systematic Reviews and Meta-Analyses) guidelines for systematic reviews and meta-analyses [27].

### Review Question

This systematic review and meta-analysis attempts to answer the following research questions: (1) What is the effect of different metal oxide nanoparticles on the strength (impact, compressive, flexural, tensile, transverse strength, and fracture toughness) of heat-cured denture base resins? (2) What is the optimum size and concentration of addition of metal oxide nanoparticles to achieve improved impact, compressive, flexural, tensile, transverse strengths, and fracture toughness of heat-cured denture base resins? and (3) Do methodological factors, such as the treatment of nanoparticles and following testing and sample fabrication guidelines, influence the strength of denture base resins?

### Eligibility Criteria

**Figure 1:**
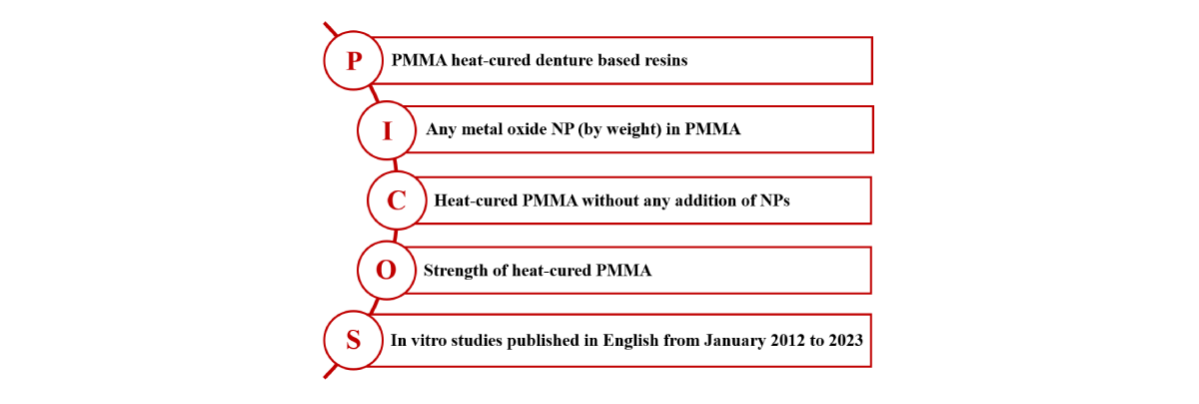
PICOS (Population, Intervention, Comparison, Outcomes, and Study design) framework for eligibility criteria. NP: nanoparticle; PMMA: polymethyl methacrylate.

The eligibility criteria of studies for inclusion are defined using the PICOS (Population, Intervention, Comparison, Outcomes, and Study design) criteria (Figure 1).

In Figure 1, population (P) denotes PMMA heat-cured denture base resins. Studies using veined, autoclaved, or microwave-cured denture base resins will be excluded. Intervention (I) denotes the addition of any metal oxide nanoparticle in PMMA only by weight. Studies based on nanotubes, fillers, fibers, coating, or hybrid nanoparticles in PMMA will be excluded. Studies that do not mention weight or volume, units of measurement, and method of mixing or dispersion of the nanoparticles will be excluded. Control (C) denotes heat-cured PMMA without any addition of nanoparticles. Outcome measure (O) denotes the strength of heat-cured PMMA. Strength was interpreted as all important parameters of strength that can affect the denture base resins, which include flexural, impact, transverse, compressive, tensile strength, and fracture toughness. Studies in which the dimensions of the samples tested were not mentioned, or if testing was done on dentures and studies in which the units of measurement were not mentioned, will be excluded. Study designs (S) denote in vitro studies published in English from January 2012 to 2023 will be considered. Thus, in vivo studies, case reports, systematic and narrative reviews, letters to the editor, short commentaries, pilot studies, or studies with preliminary results will be excluded.

### Information Sources and Search Methods for Identification of Studies

The search strategy was formulated for each database by an experienced librarian (CP) and was sent for peer reviewing to another qualified librarian using PRESS (Peer Review of Electronic Strategies) guidelines [28]. Electronic searches in 5 databases will be performed: PubMed (NCBI), Scopus (Elsevier), Cochrane Library (Wiley), CINAHL Plus with Full Text (EBSCO), and Dimensions Free Web App. Reporting of the search methods shall be done using the PRISMA-S (Preferred Reporting Items for Systematic Reviews and Meta-Analyses search extension), wherein searches will be done in databases, study registries, gray literature, and other online sources.

Other online sources that will also be searched include Google Scholar, ResearchGate, and one source for gray literature (OpenGrey). Citation chasing and manual citation searching will be done by 2 researchers (AP and AT). The details of the search strategies used can be found in Multimedia Appendix 1.

### Screening and Selection of Studies

**Figure 2:**
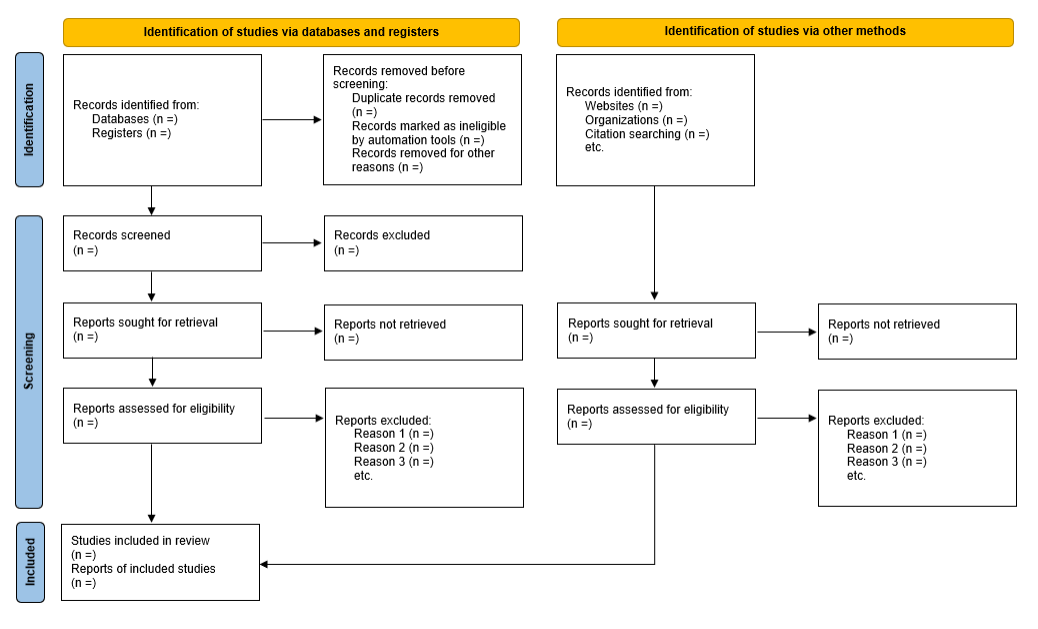
PRISMA (Preferred Reporting Items for Systematic reviews and Meta-Analyses) flow diagram to describe the study selection process.

After using the formulated search strategy in all databases and other online sources, papers or records will be imported to Rayyan (Rayyan Systems Inc) for screening. Deduplication will be performed using the same software. Two researchers (PK and AH) will independently screen the titles and abstracts of the exported papers sequentially. Full-text papers of potentially eligible studies will be retrieved to determine their final inclusion and data extraction (Figure 2 [27]). If papers are to be eliminated, the reasons for elimination will be documented.

In case of disagreements, conflicts will be resolved; however, for papers where conflicts cannot be resolved, 2 reviewers (AP and AT) will be consulted for the final decision (Figure 2).

### Process of Data Extraction

Two researchers (AP and AT) will analyze each of the included papers and extract the relevant data elements. To ensure precision, error-free, and complete extraction of data elements, extraction will be done independently. The extracted data will be tallied, and disparities will be resolved by discussions with the other 2 reviewers (PK and AH). Corresponding authors of papers will be contacted for more information or missing information in the published record if required.

### Elements of Data Extraction

Elements of data are summarized in Textbox 1. Data not stated shall be reported as **“**NS,” and unclear data shall be reported as **“**unclear.” A pilot test of the 2 researchers shall be done to ensure uniformity in understanding and procedure.

**Data elements to be extracted for the data summarization and analysis.**

**Headings and subheadings**

- Study details
  - Study title
  - Study author
  - Year of publishing
- Details of the added metal oxide nanoparticle
  - Type of nanoparticle used
  - Size of nanoparticle used
  - Concentration of nanoparticles used
  - Effect of metal oxide nanoparticle morphology on the strength
- Methods of sample fabrication
  - Treatment of nanoparticles
  - Method of dispersion of nanoparticles
  - Details of used acrylic used
  - Method of acrylization
  - Method of sample finishing
  - Storage of samples before testing
- Testing and sample details
  - Testing or sample fabrication guidelines followed
  - Sample size used
  - Sample dimensions
  - Details of testing mechanism
- Results and conclusion
  - Mean and SD of the strength tested
  - Conclusions

### Methodological and Risk-of-Bias Assessment

The assessment of the quality of the included papers will be performed using the modified CONSORT (Consolidated Standards of Reporting Trials) guidelines [29]. The risk of bias will be assessed individually by 2 researchers (PK and AH), and any disagreements will be resolved by the third reviewer (AP) based on the modified Cochrane Risk of Bias tool; scoring will be done as described in a previous study [26].

### Data Extraction

The extracted data will be presented in tabular form. The table will report the extracted variables as listed above in the *Elements of Data Extracted* section. Following data extraction, narrative as well as quantitative analyses will be performed.

### Data Analysis and Summarization

The results of all finally included studies will be described succinctly in the form of a summary table. A random-effects model will be used to calculate pooled estimates, as this is a more robust estimate regardless of heterogeneity [30]. Cochran Q and *I^2^* statistics will be used as indicators of heterogeneity. The pooled estimates of the primary end points will be calculated as the weighted mean differences with 95% CIs using the Comprehensive Meta-analysis Package (CMA version 3.0). Sensitivity analysis will be conducted to identify studies that may have severely affected the pooled estimates. Exploratory subgroup analyses by different moderator variables will also be conducted to examine sources of heterogeneity. A funnel plot and Egger linear regression test will be used to assess publication bias [31]. The significant level will be set as 2-sided and *P*<.05. Forest plots will be used to present the data.

## Results

After running the search in the 5 databases, a total of 1837 papers were found. Of these, 1752 were found to be duplicates, leaving 85 potential records that will be screened for titles and abstracts, and analyzed based on the inclusion and exclusion criteria. A similar search was conducted on other online sources, resulting in the identification of 129 papers that will be further analyzed based on inclusion and exclusion criteria. Citation chasing will be done on the finally selected papers. These numbers may slightly vary, once we update our search. The study was initiated in November 2023, where the research questions were clearly defined and search strategies were formulated. The proposed study is expected to be completed by December 2024.

**Figure 3:**
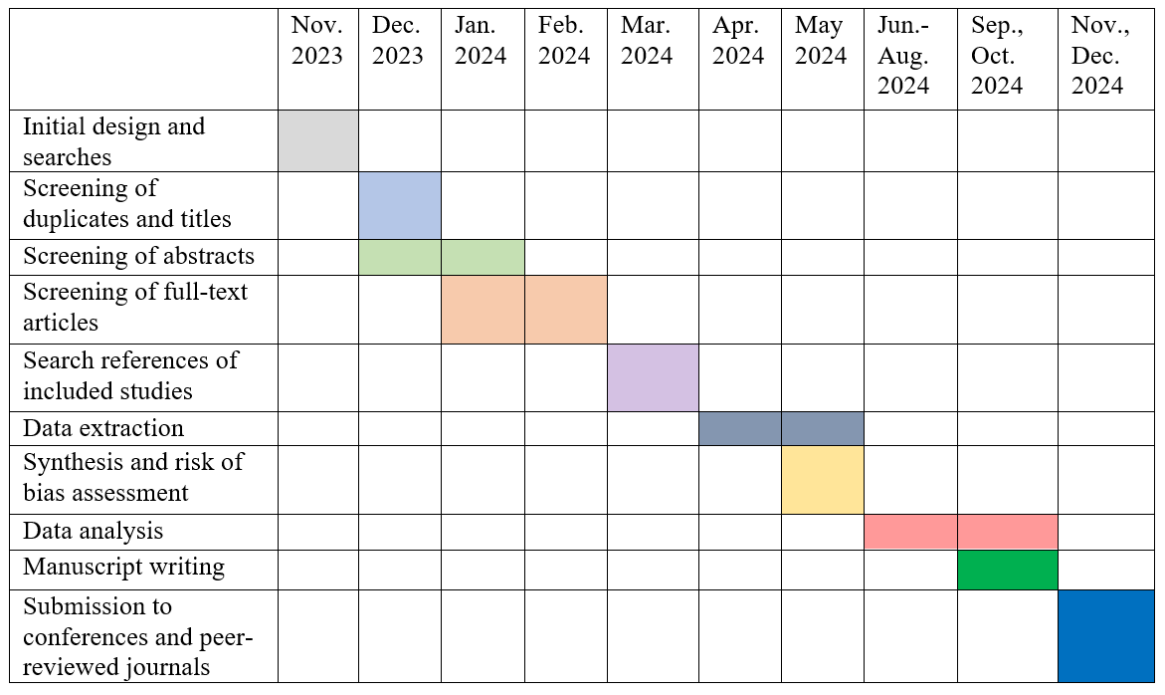
Dissemination plan of the systematic review and meta-analysis. Colored cells depict the timeline of completion of the proposed work.

The results of this systematic review and meta-analysis will be disseminated to the academic community through possible avenues, such as scientific conferences and publication in a peer-reviewed journal. A full timeline of the systematic review process is shown below in Figure 3.

## Discussion

### Principal Findings

The proposed systematic review will provide collective evidence that evaluates the effects of the addition of metal oxide nanoparticles on the different types of strength of heat-cured PMMA. It further attempts to analyze different methodological factors that can affect the strength of metal oxide nanoparticle–reinforced, heat-cured PMMA.

Previous research has shown that even though metal oxide nanoparticles can improve the mechanical properties of PMMA, they do so at certain concentrations and particle sizes [24,32-35]. Therefore, this systematic review further aims to analyze the effect of different sizes of metal oxide nanoparticles that can affect the strength. Similarly, the concentration of the nanoadditive plays a significant role in the mechanical properties, as with increased percentages, there are agglomerates formed that adversely affect the properties of the nanocomposite [23].

Other methodological factors, such as surface treatment of the nanoparticles, have also been shown to affect the properties of the nanocomposite. The use of a silane coupling agent improves the bonds between the matrix and the filler [36]. Surface treatments have also been shown to lower the surface energy of the nanoparticles, thereby preventing agglomeration or cluster formation [37]. On similar lines, following standard guidelines for testing or sample fabrication can play a crucial role in determining the robust methodology followed in the study as well as affect the results obtained. Over the years, an optimized methodology has been followed by standard organizations across countries [38].

With the advances in nanotechnology, there are several types of metal oxide nanoparticles being used and tested for their effects. To the best of the authors’ knowledge, no systematic review has attempted to evaluate and compare the effect of these different metal oxide nanoparticles on the different strengths (flexural, impact, transverse, compressive, tensile strength, and fracture toughness) of the heat-cured PMMA. We anticipate determining the optimum metal oxide nanoparticle addition conditions to heat-cured PMMA to achieve the most favorable mechanical properties about strength.

The results of the review can be used to fabricate heat-cured dentures reinforced with metal oxide nanoparticles with improved mechanical properties, subsequently improving the longevity of the dentures reducing fractures and deformation. The results of this review would also generate relevant consideration for future research to improve the quality of DBM used and continued improved quality of life for patients with dentures.

### Strengths and Limitations

The strength of the review is in the rigorous methodology that will be followed. To enhance the likelihood of locating relevant papers on this topic, a medical librarian was involved in developing a thorough search strategy. Second, robust inclusion and exclusion criteria will be followed, which will also ensure scientific rigor in answering the specific research question. Finally, all variables that can affect the strength of nanoparticle-reinforced PMMA will be analyzed either qualitatively or quantitively.

This systematic review will have some limitations that should be taken into consideration. The studies chosen for this systematic review and meta-analysis may exhibit a degree of heterogeneity due to variations in the acrylic used and the strength testing techniques. This review is limited to heat-cured PMMA resins only and does not consider other types of resins such as light-cured, autopolymerizing resin; 3D printed resins; and computer-aided design and computer-aided manufacturing denture materials. This review is restricted to pure metal oxide nanoparticles and does not consider hybrid nanoparticles. This review will not include studies published in languages other than English. Although an exhaustive literature search was performed, there may be a possibility of missing literature pertinent to the research question, due to the vast measure of articles published on this topic.

### Conclusions

This systematic review will analyze the impact of the incorporation of metal oxide nanoparticles on the different types of strength of heat-cured denture base resins. This review shall determine the optimal size and concentration of metal oxide nanoparticles to enhance the strength of heat-cured denture base resins. This study may also provide insights into the optimal methodology for adding metal oxide nanoparticles, such as following guidelines and surface treatments of the nanoparticles, to enhance the strength of heat-cured denture base resins.

## Appendix

Multimedia Appendix 1 Search strategies used in different databases.

## Abbreviations

CONSORT: Consolidated Standards of Reporting Trials
CuO: copper oxide
DBM: denture base material
OSF: Open Science Framework
PICOS: Population, Intervention, Control and Outcome Study
PMMA: polymethyl methacrylate
PRESS: Peer Review of Electronic Search Strategies
PRISMA: Preferred Reporting Items for Systematic Reviews and Meta-Analyses
PRISMA-S: Preferred Reporting Items for Systematic Reviews and Meta-Analyses search extension
SiO2: silicon dioxide
TiO2: titanium dioxide
ZnO: zinc oxide
ZrO2: zirconium oxide

## Data Availability

Data sharing is not applicable to this paper, as no datasets were generated or analyzed during this study.
