# Supplementary material for: Effect of Addition of Metal Oxide Nanoparticles on the Strength of Heat-Cured Denture Base Resins: Protocol for Systematic Review and Meta-Analysis of In Vitro Studies
Source: JMIR Res Protoc. 2024 Sep 25;13:e59999. doi: 10.2196/59999 (PMC11464938; doi:10.2196/59999)
Supplement: Multimedia Appendix 1 [file resprot_v13i1e59999_app1.docx]

**Table S1.**

| **DATABASE** | **SEARCH STRING** |
| --- | --- |
| PubMed (NCBI) | ("methylmethacrylate"[mesh] OR "methylmethacrylates"[mesh] OR "polymethyl methacrylate"[mesh] OR "PMMA"[tw] OR "PMMAs"[tw] OR "poly methyl methacrylate*"[tw] OR "polymethyl methacrylate*"[tw] OR "polymethylmethacrylate*"[tw] OR (("denture bases"[mesh] OR dental*[tw] OR "denture*"[tw]) AND ("resin"[tw] OR "resins"[tw] OR "acrylic"[tw] OR "acrylics"[tw]))) AND ("heat"[tw] OR "heated"[tw]) AND ("aluminum oxide"[mesh:noexp] OR "disilver oxide" [Supplementary Concept] OR "magnesium oxide"[mesh] OR "metal nanoparticles"[mesh] OR "nanoparticles"[mesh:noexp] OR "silver compounds"[mesh:noexp] OR "silver"[mesh]  OR "titanium dioxide" [supplementary concept] OR "zirconium oxide" [supplementary concept] OR "zinc oxide"[mesh] OR "aluminum oxide*"[tw] OR "copper oxide*"[tw] OR "cupric oxide*"[tw] OR "cuprous oxide*"[tw] OR "disilver oxide*"[tw] OR "magnesium oxide*"[tw] OR "nano particle*"[tw] OR "nano reinforc*"[tw] OR "nanoparticle*"[tw] OR "nanoreinforc*"[tw] OR "nanomaterial*"[tw] OR "nano material*"[tw] OR "silicon dioxide*"[tw] OR "silicon oxide*"[tw] OR "silver oxide*"[tw]  OR "silver"[tw] OR "titanium dioxide*"[tw] OR "titanium dioxide*"[tw]  OR "zinc oxide*"[tw] OR "zirconium oxide*"[tw] OR "zirconia*"[tw] OR "AlO2"[tw] OR "Al2O"[tw]OR "AGNP"[tw] OR "AG20"[tw] OR "MGO"[tw] OR "SIO2"[tw] OR "SIO 2"[tw] OR "TIO2"[tw] OR "TiO 2"[tw] OR "ZNP"[tw] OR "ZNPs"[tw] OR "ZRO2"[tw] OR "ZRO 2"[tw]) |
| Cochrane Library | ([mh "methylmethacrylate"] OR [mh "methylmethacrylates"] OR [mh "polymethyl methacrylate"] OR "PMMA":ti,ab,kw OR "PMMAs":ti,ab,kw OR "poly methyl methacrylate*":ti,ab,kw OR "polymethyl methacrylate*":ti,ab,kw OR "polymethylmethacrylate*":ti,ab,kw OR (([mh "denture bases"] OR dental*:ti,ab,kw OR "denture*":ti,ab,kw) AND ("resin":ti,ab,kw OR "resins":ti,ab,kw OR "acrylic":ti,ab,kw OR "acrylics":ti,ab,kw))) AND ("heat":ti,ab,kw OR "heated":ti,ab,kw) AND ([mh ^"aluminum oxide"] OR [mh "magnesium oxide"] OR [mh "metal nanoparticles"] OR [mh ^"nanoparticles"] OR [mh ^"silver compounds"] OR [mh "silver"] OR [mh "zinc oxide"] OR "aluminum oxide*":ti,ab,kw OR "copper oxide*":ti,ab,kw OR "cupric oxide*":ti,ab,kw OR "cuprous oxide*":ti,ab,kw OR "disilver oxide*":ti,ab,kw OR "magnesium oxide*":ti,ab,kw OR "nano particle*":ti,ab,kw OR "nano reinforc*":ti,ab,kw OR "nanoparticle*":ti,ab,kw OR "nanoreinforc*":ti,ab,kw OR "nanomaterial*":ti,ab,kw OR "nano material*":ti,ab,kw OR "silicon dioxide*":ti,ab,kw OR "silicon oxide*":ti,ab,kw OR "silver oxide*":ti,ab,kw  OR "silver":ti,ab,kw OR "titanium dioxide*":ti,ab,kw OR "titanium dioxide*":ti,ab,kw  OR "zinc oxide*":ti,ab,kw OR "zirconium oxide*":ti,ab,kw OR "zirconia*":ti,ab,kw OR "AlO2":ti,ab,kw OR "Al2O":ti,ab,kw OR "AGNP":ti,ab,kw OR "AG20":ti,ab,kw OR "MGO":ti,ab,kw OR "SIO2":ti,ab,kw OR "SIO 2":ti,ab,kw OR "TIO2":ti,ab,kw OR "TiO 2":ti,ab,kw OR "ZNP":ti,ab,kw OR "ZNPs":ti,ab,kw OR "ZRO2":ti,ab,kw OR "ZRO 2":ti,ab,kw) |
| CINAHL Plus with Full Text | (MH "Methylmethacrylates" OR "PMMA" OR "PMMAs" OR "poly methyl methacrylate*" OR "polymethyl methacrylate*" OR "polymethylmethacrylate*" OR ((dental* OR "denture*") AND ("resin" OR "resins" OR "acrylic" OR "acrylics"))) AND ("heat" OR "heated") AND ("aluminum oxide*" OR "copper oxide*" OR "cupric oxide*" OR "cuprous oxide*" OR "disilver oxide*" OR "magnesium oxide*" OR "nano particle*" OR "nano reinforc*" OR "nanoparticle*" OR "nanoreinforc*" OR "nanomaterial*" OR "nano material*" OR "silicon dioxide*" OR "silicon oxide*" OR "silver oxide*"  OR "silver" OR "titanium dioxide*" OR "titanium dioxide*"  OR "zinc oxide*" OR "zirconium oxide*" OR "zirconia*" OR "AlO2" OR "Al2O"OR "AGNP" OR "AG20" OR "MGO" OR "SIO2" OR "SIO 2" OR "TIO2" OR "TiO 2" OR "ZNP" OR "ZNPs" OR "ZRO2" OR "ZRO 2") |
| Dimensions Free Web App | ("methylmethacrylate" OR "methylmethacrylates" OR "polymethyl methacrylate" OR "PMMA" OR "PMMAs" OR "poly methyl methacrylate" OR "polymethyl methacrylate" OR "polymethylmethacrylate" OR (("denture bases" OR dental OR "denture") AND ("resin" OR "resins" OR "acrylic" OR "acrylics"))) AND ("heat" OR "heated") AND ("aluminum oxide" OR "disilver oxide" OR "magnesium oxide" OR "metal nanoparticles" OR "nanoparticles" OR "silver compounds" OR "silver" OR "titanium dioxide" OR "zirconium oxide" OR "zinc oxide" OR "aluminum oxide" OR "copper oxide" OR "cupric oxide" OR "cuprous oxide" OR "disilver oxide" OR "magnesium oxide" OR "nano particle" OR "nano reinforc" OR "nanoparticle" OR "nanoreinforc" OR "nanomaterial" OR "nano material" OR "silicon dioxide" OR "silicon oxide" OR "silver oxide" OR "silver" OR "titanium dioxide" OR "titanium dioxide" OR "zinc oxide" OR "zirconium oxide" OR "zirconia" OR "AlO2" OR "Al2O"OR "AGNP" OR "AG20" OR "MGO" OR "SIO2" OR "SIO 2" OR "TIO2" OR "TiO 2" OR "ZNP" OR "ZNPs" OR "ZRO2" OR "ZRO 2") |
| SCOPUS | TITLE-ABS-KEY (methylmethacrylate* OR {PMMA} OR {PMMAs} OR "poly methyl methacrylate*" OR "polymethyl methacrylate*" OR "polymethylmethacrylate*") OR (denture* OR {dental}) AND (resin* OR acrylic*) AND TITLE-ABS-KEY ({heat} OR {heated}) AND ({heat} OR {heated}) AND TITLE-ABS-KEY ({aluminum oxide} OR {disilver oxide} OR {magnesium oxide} OR "metal nanoparticles*" OR {nanoparticles} OR {silver compounds} OR {silver} OR "titanium dioxide*" OR "zirconium oxide*" OR "zinc oxide*" OR "aluminum oxide*" OR "copper oxide*" OR "cupric oxide*" OR "cuprous oxide*" OR "disilver oxide*" OR "magnesium oxide*" OR "nano particle*" OR "nano reinforc*" OR "nanoparticle*" OR "nanoreinforc*" OR "nanomaterial*" OR "nano material*" OR "silicon dioxide*" OR "silicon oxide*" OR "silver oxide*"  OR {silver} OR "titanium dioxide*" OR "titanium dioxide*"  OR "zinc oxide*" OR "zirconium oxide*" OR zirconia* OR {AlO2} OR {Al2} OR {AGNP} OR {AG20} OR {MGO} OR {SIO2} OR {SIO 2} OR {TIO2} OR {TiO 2} OR {ZNP} OR {ZNPs} OR {ZRO2} OR {ZRO 2}) |
